# Specific, situated, intra-individual, ambivalent, and open: integrating and advancing the research on entrepreneurial passion

**DOI:** 10.3389/fpsyg.2025.1453625

**Published:** 2025-04-10

**Authors:** Julia Moeller

**Affiliations:** ^1^Department of Education, Leipzig University, Leipzig, Germany; ^2^Department of Education, University of Erfurt, Erfurt, Germany

**Keywords:** entrepreneurial passion, facets, network models, situation-specific measures, intra-individual analyses, ambivalent motivation, open science

## Abstract

While the research on passion for entrepreneurial activities has produced many definitions, measures, and models specifying components, predictors, and outcomes of the construct, integrating these disparate approaches with each other and with current developments in psychological science remains a challenge for the next generation of research studies. This review connects the research on entrepreneurial passion with current innovations and debates in measurement and method development, motivation, personality, and developmental psychology. The review proposes to reconsider how to measure entrepreneurial passion by (1) specifying the exact facets of entrepreneurial passion in theoretical models and measures, and (2) using psychometric and co-endorsement network models to examine the relationships among the facets and the facets’ relationships with relevant predictors and outcomes. (3) The article proposes to link passion research to ongoing debates about states, traits, and emerging stability by formulating and testing process models that distinguish between state- and trait development and include recursive feedback loops. (4) The review connects research on entrepreneurial passion to current debates in emotion and motivation research by proposing to examine the ambivalent motivation and mixed emotions accompanying entrepreneurial passion with intra-individual methods. (5) To help passion researchers build on each other’s work and enhance the trustworthiness of their work, the review calls for cumulative scientific insights by adopting multi-lab collaborations and other open science practices. (6) Finally, the review proposes a new, integrative theoretical model that distinguishes between the facets that drive affective thriving and those driving perseverance in the face of obstacles.

*“(…) there is no such thing as a life of passion*

*any more than a continuous earthquake, or an eternal fever.*

*Besides, who would ever shave themselves in such a state?”*

*(Lord Byron, July 5th, 1821/1833)*

## Introduction

In two decades, the research on passion, including entrepreneurial passion, has produced various definitions, measures, and models specifying components, predictors, and outcomes of the construct (for reviews, see, e.g., Iyortsuun et al., 2019; Lee and Herrmann, 2021; Moeller, 2014; Murnieks et al., 2020; Newman et al., 2019; Riar et al., 2023; Schwarte et al., 2023; Thongmanivong, 2020; Zhao and Liu, 2023). A challenge for the next generation of research is the integration of these diverse theoretical and methodological approaches with each other and with other relevant research fields. This conceptual article seeks to contribute to such an integration. It aspires to reconcile some core insights from various passion models with insights from other relevant research fields, including the literature on related motivation and emotion constructs, the literature on relevant measurement and methodological issues, and the literature on states, traits, and development.^1^

The article consists of two main parts, the first of which summarizes open questions in the previous literature and the second makes suggestions how to address these gaps.

## Open questions in the current literature on passion

This first section summarized open questions in the previous research on entrepreneurial passion. Table 1 gives an overview about these open questions summarized in section 1 and the proposed solutions suggested in section two.

**Table 1:** Overview of open questions and proposed solutions.

Open question (summarized in section 1)	Proposed solutions (suggested in section 2)
1. There is no consensus about the facets of passion. Most models agree in conceptualization passion as a multi-facetted construct, but many do not distinguish between the specific facets in measurement instruments and there is no consensus across models as to which facets to include or exclude in definitions and measures of entrepreneurial passion.	1. Define and measure the specific facets of passion. Compare them with facets of related constructs. Estimate relations with predictors, correlates and outcomes on the level of specific facets, as they may differ between them. Examine the development of facets separately.
2. There has not yet been a systematic debate about appropriate measurement models for passion, and the currently frequently used reflective latent variable models make many assumptions that are known to be unrealistic for multifaceted constructs, including passion.	2. Test the assumptions of the planned measurement model. If the assumptions are not supported by data or theory, use a different measurement model. Consider alternative measurement models than the dominating reflective latent variable models. For instance, describe the associations concurrent or lagged associations among specific facets of passion with psychometric, zero-correlation, and co-endorsement network models.
3. The role of state- versus trait facets of passion is unclear. The stable components of passion (e.g., identification, long-term goals, perseverance) tend to be emphasized more pronouncedly in several models, but these models nevertheless also include components and correlates of passion that are known to fluctuate, such as emotions and flow experiences.	3. Examine entrepreneurial passion with process models. Consider that different facets of passion can develop on different timelines and examine their development separately to test this possibility.
4. The role of positive and negative emotions in passion is unclear. Some models include only positive emotions. Others emphasize the difference between positive (harmonious) and negative (obsessive) aspects of passion, but leave open whether these describe distinct types of individuals with different profiles of HP and OP, or if HP and OP co-occur within individuals	4. Connect research on entrepreneurial passion with research on mixed emotions and ambivalent motivation. A multi-facetted construct can include both positive and negative facets. A person can experience both positive and negative emotions and aspects of motivation. There are theories describing such mixed emotion and ambivalent motivation, and there are methods that are capable of revealing such mixed feelings and ambivalence, unlike the currently dominating between-person structural equation models, which mostly overlook such ambivalence (see point 5).
5. Intra-individual methods are needed but largely missing in the literature on passion for entrepreneurial and other activities.	5. Use intra-individual methods, such as latent profile analyses, co-endorsement networks, or intra-individual analyses of intensive longitudinal data to examine within-person patterns of passion facets and their predictors and outcomes. This may be crucial to the trustworthiness of research on entrepreneurial passion.
6. Definitions, measures and studies on passion for entrepreneurial and other activities are studied in mostly isolated silos that fail to build on each other.	6. To move the field forward, build on each other, increase transparency, replicability and generalizability, collaborate across research teams, across research lines (different models, measures), and across disciplines. Adopt open science practices to facilitate such cooperation.

By integrating insights from different, mostly separated lines of research on passion, related motivational constructs, personality psychology and current methodological debates, this article aims to make contributions to answering the following research questions: (1) What exactly are the facets of the multi-facetted construct of entrepreneurial passion? (2) What are appropriate measurement models for measuring facets of (entrepreneurial) passion and the associations among them? (3) How does entrepreneurial passion differ from related constructs? What does entrepreneurial passion contribute to the prediction of relevant outcomes beyond the contributions made by other constructs (incremental validity)? Which specific passion facets account for such incremental validity in predicting which outcome? (4) How do malleable, fluctuating aspects of entrepreneurial passion interact with stable components? Which of these components are linked to which outcomes and which predictors? (5) How can we reconcile that some studies consider positive and negative experiences as rather mutually exclusive, while others report co-occurrences of positive and negative experiences in the same passionate individuals? (6) How can we integrate the calls for more intra-individual methods in the recent psychological literature into the research on passion, and what advancements do we expect to gain from these novel methods? (7) What definition, measurement model, and study design could integrate the insights from the afore-mentioned points, and the insights from the various approaches in the previous research, in a joint framework? All of these questions are explained more in detail below, before conceptual and methodological approaches jointly addressing them all are introduced.

### What is entrepreneurial passion? A multifaceted construct with sometimes fuzzy boundaries

There are many different definitions and measurement instruments of passion and it is beyond the scope of this article to give a comprehensive overview of all of them. Instead of attempting such as all-encompassing integration, this article aims to integrate some core components of a few of the so far most influential models, which have previously been studied mostly in isolated silos. This manuscript proposes a joint framework for the integration of these few selected lines of research on (entrepreneurial) passion, but remains open for future amendments to integrate key insights from further passion models not addressed here. When summarizing research gaps in the previous literature and proposing approaches to integrate core elements of various models, this article will rely mainly on the research on the dual model of passion (e.g., Vallerand et al., 2003; Vallerand and Houlfort, 2019), aspects of entrepreneurial passion (e.g., Cardon et al., 2009; for an overview, see Newman et al., 2019), research distinguishing between malleable and stable aspects of passion for work (Chen et al., 2015; O’Keefe et al., 2018; Duckworth et al., 2007; Jachimowicz et al., 2018a) and models distinguishing between specific facets of passion (Moeller, 2014; Moeller et al., 2019). By no means is that supposed to be a complete list of insightful lines of research on passion, it is merely a first set of rather isolated lines of research which to integrate will be beneficial for future research on entrepreneurial passion.

Vallerand et al. (2003) emphasize four core components of passion (liking an activity, investing time and energy into that activity, calling the activity a passion and identifying with the activity). The authors furthermore distinguish between two correlated facets of passion, namely the beneficial harmonious passion and the harmful obsessive passion. While Vallerand et al. (2003) and many of the studies using their dual model of passion treat passion as a domain-unspecific form of motivation that can be measured with the same items no matter what activity it refers to, Cardon et al. (2009) focus on only entrepreneurial passion and have identified different roles of the passionate entrepreneur and different types of entrepreneurial activities a person can be passionate about (e.g., Cardon et al., 2017). Unlike Vallerand et al. (2003), who include both positive and negative emotions as correlates and outcomes of passion, the entrepreneurial passion concept by Cardon et al. (2009) emphasizes mostly the positive emotions accompanying entrepreneurial passion. Both authors leave it a bit open whether passion is a stable trait-like disposition (or inclination, as Vallerand et al., 2003 call it), or if passion includes malleable, state-like components (as their inclusion of emotions and flow experiences suggests, which are known to vary within short periods between situations and contexts). This question about state and trait components has been addressed in studies by Chen et al. (2015), O’Keefe et al. (2018), Duckworth et al. (2007), and Jachimowicz et al. (2018b). While Duckworth et al. (2007) have emphasized the stable or stability-driving components of passion, others have studied the components of passion that can be changed (e.g., Jachimowicz et al., 2018b; Moeller et al., 2017; Schwarte et al., 2023). This article proposes to integrate both aspects into a joint framework by acknowledging that passion is a multifaceted construct that includes both malleable, state-like and stable, trait-like facets that need to be disentangled and understood in process models that describe their different timelines and contributions to experiences, behaviors, and outcomes of entrepreneurial passion.

Most models and measures of entrepreneurial passion and passion in general include a number of passion facets and measure passion as a composite (either an average, or latent variable) of various facets (for overviews, see, e.g., Iyortsuun et al., 2019; Lee and Herrmann, 2021; Moeller, 2014; Newman et al., 2019; Riar et al., 2023; Schwarte et al., 2023; Thongmanivong, 2020; Zhao and Liu, 2023). Conceptualizing passion as a multi-facetted construct enables us to examine the nature of entrepreneurial passion more in-depth by addressing research questions, which are summarized along with their implications for researchers and practitioners in Table 2.

**Table 2:** Practical implications of open questions regarding facets of entrepreneurial passion.

Questions about facets of entrepreneurial passion	Implications of these questions for researchers	Implications of these questions for practitioners
1. Which of these facets need to be experienced by a person for this person to be labeled passionate?	This question must be answered for researchers to be able to identify passionate individuals. So far, there is no consensus and much controversy about this question among researchers.	Managers, business angels, or founders selecting partners can only identify passionate entrepreneurs and employees if it is clear what exactly indicates a passion.
2. Can one facet’s presence compensate for the absence of another for a person to still be labeled passionate?		
3. What would be an appropriate measurement model to describe the relationship among these facets in a passionate person?	Only if passion is measured well, researchers can draw valid conclusions. Good measurement would help solving the measurement crisis (Flake and Fried, 2020)	
4. Which exact facet accounts for which outcome or correlate of passion? Which predictor affects which facet? What are the psychological *mechanisms* and processes behind these statistical relationships among the passion facets and the predictors, correlates, and outcomes of passion?	Understanding mechanisms linking passion to predictors and outcomes can help developing interventions aiming to increase -or decrease- passion in general and passion facets in particular. Associations of specific facets to predictors and outcomes tend to be easier to interpret and higher than associations of higher-order factors to predictors and outcomes.	An entrepreneur who wants to increase their own passion, or a manager aiming to increase the passion in their employees, needs to know what influences which aspect of passion. Managers and entrepreneurs may want to know which exact aspects of passion drive which outcomes, to focus their efforts on the aspects linked to the most relevant outcomes.
5. In what intra-individual combinations are these facets experienced by individuals with an entrepreneurial passion?	The validity of conclusions about predominantly harmonious and predominantly obsessive passionate entrepreneurs depends on the question whether intra-individual person-oriented methods confirm that there are these distinct types of individuals with distinct intra-individual patterns of HP and OP. That is unclear, as most previous studies only use inter-individual variable-oriented analyses. A core assumption of previous research in therefore questionable and needs confirming	Only if intra-individual patterns are studied, different types of passionate entrepreneurs (e.g., predominantly harmonious versus predominantly obsessive) entrepreneurs can be identified. Given that previous research suggests that these groups -if they exist- may have very different needs for support, risks and strengths, appropriate counseling requires this distinction and therefore assessments relying on intra-individual analyses.
6. In which facets does entrepreneurial passion overlap with related constructs, such as work engagement and commitment, and are there any facets, or combinations thereof, that are unique to the passion construct?	Identifying overlaps across multifaceted constructs would enable researchers to shorten assessment instruments by avoiding to repeatedly assess redundant facets.	People having their passion assessed may wish for that assessment to be economic, i.e., not wasting their time.
7. Does the passion construct have incremental validity in explaining relevant work experiences and entrepreneurial outcomes, compared to related constructs? This question needs to be examined on the level of specific facets, raising the additional question of which facets account for that incremental validity of passion in comparison to which competing constructs and which outcomes.	Incremental validity concerns jingle and jangle fallacies. If passion -or a part of it- is just a new synonym for a facet known under a different name, then researchers from these different lines of research fail to build on each other. Research resources are wasted. Research contributes to confusion rather than clarification. Since correlations with outcomes tend to be stronger and easier to interpret in the level of facets than factors,	Practitioners need effective measures and interventions. If there are constructs that predict relevant outcomes better than others, it might save practitioners time and money focusing in the constructs with the better predictive and incremental validity.
8. Which facets develop at which time line (stable versus fluctuating facets)?	Answering this question would inform process models of entrepreneurial passion, which remain scarce.	If an entrepreneur wishes to be perceived as passionate to business angels, a manager wants to increase positive experiences or perseverance, focusing on what is malleable makes the effort more effective.

As the points four, five, and seven above show, the distinction between specific facets of passion is an important step toward a better understanding of the relationship of passion to other constructs. Many definitions of entrepreneurial passion overlap so much with other constructs (e.g., work engagement, commitment, personal interest) that it is often unclear where one construct begins and the other ends (for overviews, see Moeller, 2014; Newman et al., 2019). Consequently, there are multiple labels referring to similar phenomena, which is called a jangle fallacy (Block, 1995), and many different definitions of entrepreneurial passion describing slightly different phenomena, called a jingle fallacy (ibid.). Both jingle and jangle fallacies make it difficult for researchers to know what exactly someone means when they use the term passion. This hinders researchers from building upon previous research in a cumulative way, because it is often unclear if previous studies addressing entrepreneurial passion actually referred to the same phenomenon. Unclear boundaries of passion to related constructs in combination with jingle and jangle fallacies also prevent the integration of insights from other motivational constructs into the research on entrepreneurial passion, because it is unclear in which aspects passion overlaps with similar constructs, such as engagement, commitment, or emotions.

### How should we measure entrepreneurial passion? What are appropriate measurement models to represent the relationships among its indicators and facets?

In addition to the different definitions and models, there are various measures of passion with relevance to research on entrepreneurial motivation, including the dual model passion scale (Vallerand et al., 2003; Ho et al., 2018), measures of entrepreneurial passion (for an overview, see Newman et al., 2019; Zhao and Liu, 2023), measures of lay people’s beliefs about passion for work (Chen et al., 2015; O’Keefe et al., 2018; Jachimowicz et al., 2018b), and facet measures of work passion (e.g., Moeller et al., 2019). To reduce confusion due to jingle and jangle fallacies and to build upon each other’s work in a cumulative way, it seems desirable to strive toward some agreement about how to measure entrepreneurial passion.

The first step toward that goal would be the agreement about facets and items to be included in measures of entrepreneurial passion. The next step would be the identification of an appropriate measurement model. There have been recent debates in the motivation and emotion literature about the features of different measurement models. It has been pointed out that the commonly used structural equation models have strict underlying assumptions that may be unrealistic and in conflict with theoretical assumptions of theories about multifaceted motivational constructs (e.g., schoolwork engagement; see Kulakow and Moeller, 2024; success expectancies and task values; see Jähne et al., 2024; Moeller et al., 2022), as well as emotions (e.g., Lange et al., 2020; Haslbeck et al., 2019). We will discuss below if the same critique applies to measures of entrepreneurial passion, which has relied mostly on such structural equation models, and whether alternative measurement models might be more realistic and in harmony with theoretical assumptions about the relationships among passion items and passion facets.

So far, most studies have measured overall passion or its facets (e.g., harmonious and obsessive passion), in form of a composite (average score or latent variable). To justify these composite measurements, many studies refer to findings of acceptable model fit indices in confirmatory factor analyses (CFA; e.g., Jachimowicz et al., 2018b; Vallerand et al., 2006; Vallerand, 2010). While CFA are frequent in psychology and motivation research, they are not automatically the most appropriate measurement models (e.g., Lange et al., 2020; Kulakow and Moeller, 2024). The passion research has not yet seen any systematic debate about appropriate measurement models for the passion construct, nor a debate about the advantages or disadvantages of latent variable models. However, the research on passion can benefit and gain insights from the debates about suitable measurement models for other multifaceted constructs of motivation, which have recently concluded that systematic comparisons of latent variable models (e.g., CFA) with psychometric networks,^2^ and zero-order correlation networks,^3^ co-endorsement networks,^4^ are needed to identify the best measurement model and to reveal eventual violations against the crucial assumptions that the typically used latent variable models rely on.

**Figure 1:**
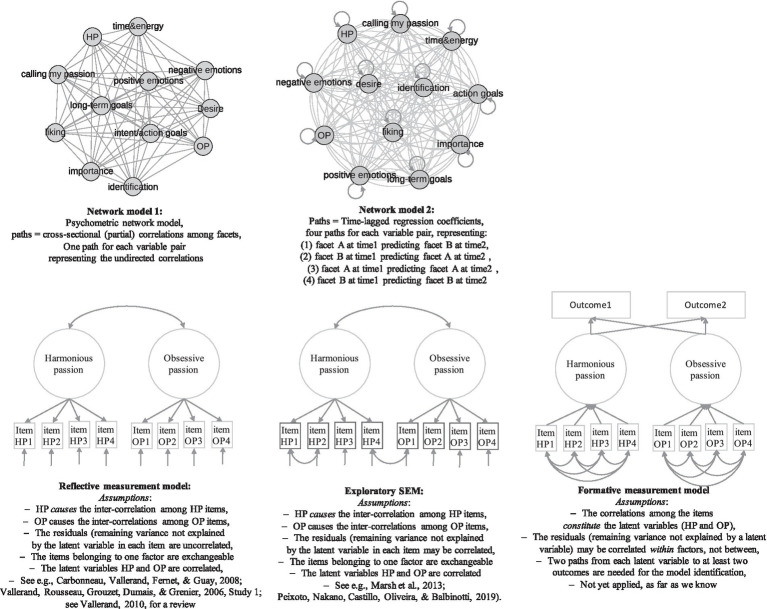
Measurement models representing the *structure* of passion. Please note that the facets shown here are only examples, derived from only two models of passion (Vallerand et al., 2003; Moeller, 2014). In addition to these facets, these measurement models can be applied for different sets of passion facets in future studies.

The frequently used confirmatory factor analyses have strict assumptions (e.g., common-cause relation, separate identifiability, local homogeneity, local independence, assumption of exchangeability of all indicators; for definitions and discussions, see, e.g., Lange et al., 2020; Kulakow and Moeller, 2024). Many of these assumptions have been found to be unrealistic and empirically unsupported in studies on other multifaceted motivation constructs and studies on emotions (Lange et al., 2020; Jähne et al., 2024; Kulakow and Moeller, 2024). There are several alternative measurement models with less restrictive and vastly different implications for the structure of passion items and facets that, as far as we know, seem not to have been considered yet by the passion literature (see Figure 1). In addition, there are various statistically equivalent models that may show the same model fit but imply very different conclusions about the inter-relations and causal dependencies of the examined items or facets (see, e.g., Brick and Bailey, 2020; Lange et al., 2020; Tomarken and Waller, 2005). Figure 1 gives an overview of some possible measurement models, with the lower left model (the so-called *reflective measurement model*, also called CFA or confirmatory SEM) being the most frequently applied in the research on passion.

There are several reasons to consider measuring entrepreneurial passion with other than the commonly used reflective measurement model: first, the strict underlying assumptions underlying the reflective measurement model (i.e., CFA) are likely unrealistic, for a multi-facetted construct such as passion (see Lange et al., 2020; Jähne et al., 2024; Kulakow and Moeller, 2024). For instance, the reflective measurement model assumes that the latent variable represents *one mechanism or entity causing* all the inter-correlations among the indicators or facets, which may seem implausible.

Moreover, the reflective measurement model, i.e., CFA, assumes that the residuals (variance component in each item not explained by the latent factor) are uncorrelated, which several studies on the dual model passion scale found to be unsupported by empirical evidence (Marsh et al., 2013; Peixoto et al., 2019). A few studies have therefore suggested to loosen the constraint of uncorrelated residuals in exploratory structural equation models (ESEM; see Figure 1) allowing correlations among the residuals, both within and across the indicators of harmonious and obsessive passion (ibid.). This decision, however, should give rise to a broader debate about the appropriate measurement model used to represent entrepreneurial passion. Ideally, the measurement model should be derived from the theoretical model of the construct and the use of confirmatory theory testing through model fit indices is the way to know whether the data support the theoretical structural model of passion. Now that the measurement model had to be revised due to discrepancies between the empirical structure (correlated residuals) and the theoretically assumed structure (expecting uncorrelated residuals), we should take this as a reason to review the theoretical assumptions that we have about relations among passion indicators, and the most appropriate measurement model reflecting these assumptions best. A much-needed debate about appropriate measurement models for modeling entrepreneurial passion should address for instance the following questions: do we really assume that all relations among the indicators of passion are *caused* by *one single psychological mechanism*, in passionate individuals, as the CFA assumes? If so, what is this psychological mechanism underlying this assumed latent variable? The finding of substantial correlations among the residuals of the latent factors representing harmonious and obsessive passion (Marsh et al., 2013) suggests that a measurement model with less restrictive assumptions might be more appropriate. If we do not assume that one single mechanism causes the correlations among passion indicators, we might maybe want to consider for instance a formative measurement model, in which the latent variable is constituted by what the indicators have in common, rather than being the *cause* of their shared variance.

Research on motivation is only starting to appreciate the manifold insights that network models contribute. To give examples, Moeller et al. (2018a) showed that co-endorsement networks, but not correlation-based methods were able to reveal mixed emotions in both cross-sectional and intensive longitudinal studies. Kulakow and Moeller (2024) showed how psychometric networks and co-endorsement networks revealed associations between specific facets of (schoolwork-related) engagement and burnout that the reflective latent factor models would have overlooked. Jähne et al. (2024) demonstrated the same for associations among components of task values, costs, and success expectancies, all features of the influential expectancy-value theory. All of these studies demonstrated that networks based on partial correlations, zero-correlations and co-endorsements reveal associations among facets of multi-facetted constructs that the commonly used latent variable models may overlook. That is particularly relevant for multi-facetted constructs that represent ambivalent motivation and/or mixed emotions, as Moeller et al. (2018a, 2018b) point out. Since entrepreneurial passion is both theoretically and empirically such an ambivalent experience accompanied by co-occurring positive and negative feelings, we can expect that methods capable of revealing such co-occurrences (i.e., co-endorsement networks and cluster/latent profile analyses) are crucial to the understanding of such motivational ambivalence.

### State- versus trait facets of passion

While previous research has included many fluctuating constructs, such as state emotions and flow, as facets and correlates of passion, there is a lack of empirical longitudinal studies capturing such states and their fluctuations across entrepreneurial work situations. The available intensive longitudinal studies needed to capture states at best address related constructs, such as work engagement (Riedl and Thomas, 2019), workaholism (Snir and Zohar, 2008), flow at work (Fullagar and Kelloway, 2009); or they capture situational experiences of passion in other than work contexts (e.g., in adolescents’ everyday lives, Moeller et al., 2017).

Since we know so little about people’s passionate experiences in specific entrepreneurial activities, it is unclear how the abstract, domain-unspecific multifaceted construct of passion relates to the exact experiences that people make. How exactly do people feel, think, and act, in which exact situations, if they are passionate about their entrepreneurial ventures? And how do the rather stable passion components, such as the “inclination” (Vallerand et al., 2003), the identification (e.g., Vallerand et al., 2003; Cardon et al., 2009) and the long-term goals (Moeller et al., 2019) relate to the presumably fluctuating passion components? We need intensive, as well as long-term, longitudinal studies in combination with theoretical process models describing the development of state and trait passion facets on their respective time lines, describing the psychological mechanisms of the facets’ interactions with each other, and describing the facets’ respective contributions to relevant outcomes, or unique dependencies on antecedents (for first process models, see, e.g., Yoo et al., 2023; for models specifying possible moderator effects, see, e.g., Laskovaia et al., 2022; Lee et al., 2021).

### Rethinking the relation of passion to positive and negative outcomes

Much of the research on entrepreneurial passion so far has been influenced by the dual model of passion (Vallerand et al., 2003; Vallerand and Houlfort, 2019), which often describes a person’s passion as either predominantly harmonious, or as predominantly obsessive (e.g., Vallerand et al., 2010). Other models of entrepreneurial passion have left out the negative component and define entrepreneurial passion as a mainly positive experience (e.g., Cardon et al., 2009). In contrast, several studies found that positive and aversive experiences can co-occur in passionate individuals (e.g., Moeller et al., 2015; Wang et al., 2008). This raises the question whether an entrepreneurial passion is best described as dualistic (either predominantly obsessive, or predominantly harmonious), or as an ambivalent experience (positive and negative at the same time). Recent findings of ambivalent motivation in the closely related work engagement construct suggest that substantial groups of employees report ambivalent combinations of high work motivation (engagement) co-occurring with high levels of stress and burnout symptoms (e.g., Moeller et al., 2018b). In line with this finding, we might want to consider whether the emerging literature on mixed emotions (e.g., Hoemann et al., 2017; Miyamoto et al., 2010; Moeller et al., 2018a) may provide insightful additions to our understanding of ambivalence in individuals with an entrepreneurial passion. The possibility that harmonious and obsessive aspects may co-occur is supported by the finding that their assumed precursors, intrinsic and extrinsic motivators, may co-occur within individuals (Lepper et al., 2005). In addition to theoretical models explaining such possible ambivalence in people with an entrepreneurial passion, we may want to consider process models specifying the long-term dynamics among harmonious and obsessive experiences. So far, the relationship between harmonious passion (HP) and obsessive passion (OP) was mostly considered time-invariant, since differences between HP and OP are expected to go back to how an activity was originally internalized, and such past internalization seems unlikely to change. However, due to the lack of longitudinal studies, we do not know yet whether a predominantly harmonious passion, if it exists, may turn into an OP, or vice versa, if the entrepreneurial activities, the stage of the entrepreneurial venture, or other conditions change. We may, for instance, hypothesize that changes to entrepreneurial work conditions causing goal conflicts or exhaustion may increase the obsessive and negative experiences in a formerly harmoniously passionate activity (think for instance of a startup leaving the phase in which a small team of year-long friends collaborates on eye level on a topic all of them feel passionate about, toward a phase of controlled grow, in which suddenly managers, hierarchies, and time pressure influence who works when how and on what).

### Intra-individual and person-oriented analyses

Many studies have shown that the afore-mentioned ambivalence or intra-individual co-occurrence of harmonious, positive, and obsessive, negative experiences may be overlooked if inter-individual and correlation-based methods are used to examine the relationships between these experiences. Inter-individual and correlation-based methods prevail in the vast majority of studies on passion for entrepreneurial and other activities. More intra-individual analyses of within-person co-occurrences and within-person profiles of positive and negative experiences and other facets of passion seem needed for various reasons: (1) intra-individual methods have revealed ambivalent intra-individual co-occurrences of positive and negative passion facets (see above; Moeller et al., 2015; Wang et al., 2008), and systematic replications of these findings are needed, (2) the prevailing inter-individual and correlation-based methods often overlook co-occurrences/co-endorsements of two constructs within the same individuals, because correlations or positive regression coefficients do not imply co-endorsement, and because various subgroups of individuals with widely different profiles of the correlated variables can hide behind such overall, inter-individual coefficients (e.g., Anscombe, 1973; Matejka and Fitzmaurice, 2017; Moeller, 2021), (3) studies on related constructs (e.g., work engagement, mixed emotions, expectancy-value components), indicate that the intra-individual structure and processes may differ between individuals (called lacking ergodicity), and that intra-individual methods in combination with intensive longitudinal studies are needed in order to discover such heterogeneity (e.g., Fleeson, 2004; Mischel and Shoda, 1998; Molenaar, 2004).

## Integrating diverse models and measures of entrepreneurial passion by implementing recent innovations in the research on motivation and emotions

This second section proposes novel approaches to conceptualize and measure passion that provide solutions to the open questions discussed in section one above. It seeks to integrate recent innovations and insights from the larger literature of emotion and motivation into the research on entrepreneurial passion, to help reconciling separate entrepreneurial passion definitions and measures in joint frameworks and studies.

### Specifying the exact facets of entrepreneurial passion

Acknowledging the multi-facetted structure of passion more explicitly in definitions and psychometric models of entrepreneurial passion seems helpful for the following reasons: (1) defining passion as a multi-facetted construct seems to be the minimal consensus that most, if not all, passion researchers can agree to (for overviews, see Newman et al., 2019; Moeller, 2014). (2) Defining and measuring the specific facets of passion, and of other multi-facetted constructs, enables us to delineate the differences from and overlaps with related constructs, and thus avoid jingle and jangle fallacies and supports cumulative research. (3) Correlations among specific facets generally tend to be stronger and content-wise easier to interpret than correlations among more abstract higher-order factors (e.g., Armstrong and Anthoney, 2009; Beauducel et al., 2007; Bipp et al., 2008; Schimmack et al., 2004). (4) Specifying facets enables us to distinguish between the unique contributions of different facets to relevant outcomes, and the differential dependency of different facets on different predictors. (5) Distinguishing between all relevant specific facets of passion enables us to find an appropriate measurement model. Some of these points are discussed more in detail below.

First, describing entrepreneurial passion as a multi-facetted construct is very common among researchers (Newman et al., 2019; see also Cardon et al., 2009, 2013; Adomako and Ahsan, 2022; Ahsan et al., 2023). Thus, identifying the specific facets in which two definitions, models, or measures agree, and those they do not agree about, seem important first steps toward identifying the core of entrepreneurial passion and integrating the various passion definitions and measures.

Second, specifying the exact facets of entrepreneurial passion allows us to delineate more precisely in which of these facets the passion construct overlaps with or differs from related constructs. For example, the related construct of engagement for work also involves the facets of liking, finding important, spending time and energy, and positive emotions, but unlike (obsessive) passion, engagement comes without the connotation of negative, obsessive feelings and irrational persistence (Gorgievski-Duijvesteijn and Bakker, 2010; Ho et al., 2011). On the other hand, workaholism is extremely similar to obsessive passion, both theoretically as empirically (ibid.; Birkeland and Buch, 2015). Specifying facets also helps distinguishing entrepreneurial passion from organizational and work commitment, another multi-facetted construct, which according to one review differs from passion mainly in the facet of urging approach motivation/desire, which passion includes and commitment does not (Moeller, 2014). So far, our knowledge about overlaps between entrepreneurial passion and related constructs has been predominantly empirical, with intercorrelations being reported and interpreted. Ideally, such empirical findings of partial overlaps should be preceded or at least accompanied by theoretical assumptions about the *psychological mechanisms* underlying such covariance.

The third reason to define and measure entrepreneurial passion in terms of specific facets is the insight that correlations or regression coefficients among facets are often easier to interpret than similar findings concerning relationships among higher order factors (e.g., Armstrong and Anthoney, 2009; Beauducel et al., 2007; Bipp et al., 2008; Schimmack et al., 2004). Imagine finding a moderate or high correlation among HP and OP, as it has been reported in numerous studies (e.g., Rip et al., 2006; Vallerand et al., 2007; Séguin-Lévesque et al., 2003; Vallerand et al., 2003). Both HP and OP are multi-facetted themselves, with HP including facets such as goal harmony, being enables to “live a variety of experiences,” and positive feelings; and obsessive passion including facets such as goal conflicts, addiction-like symptoms such as urge/craving, withdrawal symptoms and ill-advised persistence, and aversive feelings (e.g., Vallerand et al., 2003, p. 760). In addition, HP and OP should theoretically both include the passion criteria (liking, finding important, spending time and energy, calling one’s passion, and identifying with the activity), each of which can be considered a facet (or two facets, in the case of time—facet 1, and energy—facet 2). Thus, the moderate correlation between HP and OP could be due to them sharing these passion criteria, or due to the possible intra-individual co-occurrence among genuinely harmonious and genuinely obsessive experiences (e.g., mixed emotions), which was observed in several intra-individual studies (e.g., Moeller et al., 2015; Wang et al., 2008). Examining only the correlation among both multi-facetted sub-factors of passion makes it hard to determine which exact facets, or items, account for this correlation. In contrast, distinguishing between the more specific facets of HP and OP (e.g., goal conflicts, intrinsic experiences, opportunity for growth, addiction-like symptoms etc.) would be much easier to interpret, and possibly helpful in finding out which exact facets account for the correlation among HP and OP. That much insight can be contributed by examining the facet-level relations has been shown by research on constructs related to entrepreneurial passion, including vocational interests (Armstrong and Anthoney, 2009) and conscientiousness, of which grit – and therefore the grit facet called passion– is a facet (Schmidt et al., 2020; Ponnock et al., 2020).

**Figure 2:**
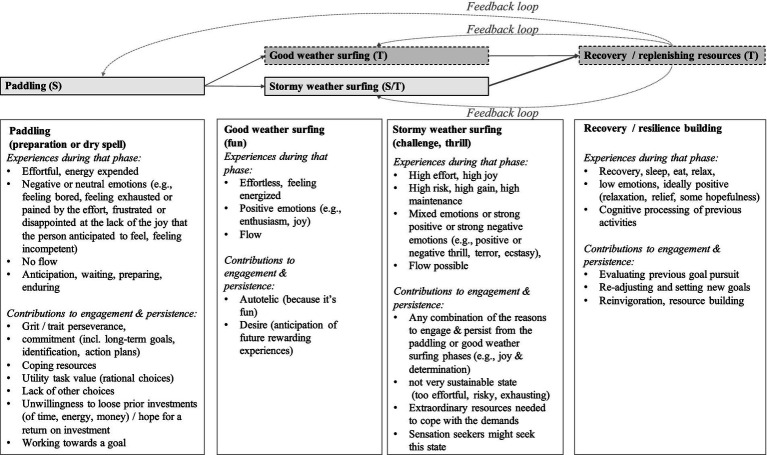
The strive (S) and thrive (T) process model of passion.

The fourth reason to distinguish between facets of entrepreneurial passion refers to the relations among passion and its predictors, correlates, and outcomes. As I point out below (e.g., Figure 2), passion facets can be classified for instance into those accounting for emotional-motivational thriving (e.g., flow, positive emotions, desire, energy, interest), and those accounting for striving and perseverance in the face of obstacles (e.g., identification, long-term goals, trait perseverance/grit, or the ability to endure suffering accompanying obsessive passion). Does it not seem plausible that different predictors would affect these facets differently? Or that different facets make different contributions to the explanation of relevant outcomes? If you were an entrepreneur with a small start-up team, a manager in a large company, or a coach in a professional sports team, would you not wish to know which exact facet is (a) lacking in your employees/athletes, (b) malleable and influenceable by your actions, and (c) relevant for the crucial desired outcome?

The fifth reason to distinguish between specific facets refers to the need to be specific in the definition in order to find the most appropriate measurement model. Items need to fit to the theoretical concept of the construct they are supposed to capture. Everyone understands roughly what is meant if the term passion is used in definitions, but under closer inspection, it often turns out that different people -including different researchers- understand and capture different things when referring to the term. Thus, clarity about specific facets is needed when developing measurement models that capture exactly what we mean by passion.

### Describing the structure of passion facets in psychometric, zero-correlation, and co-endorsement network models

If we define passion as a multi-facetted construct, we need a corresponding measurement model describing these facets, and describing the theoretical assumptions and previous empirical findings about the relationships among these facets. Such a measurement model for a multi-facetted construct like entrepreneurial passion should take into account that the inter-correlations among the different passion facets may vary among facets, meaning some facets may be stronger related to each other than others (see, e.g., Kulakow and Moeller, 2024; Jähne et al., 2024 for similar findings concerning other multi-facetted motivation constructs). A reflective latent variable model^5^ would automatically constrain the correlations among passion facets to the covariance explained by a higher-order passion factor, ruling out any residual correlations, but I see no theoretical reason justifying this assumption for entrepreneurial passion. Instead, it seems plausible – and even has been demonstrated empirically in various studies (e.g., Marsh et al., 2013) – that certain facets and single items can have residual correlations beyond their degree of covariance that is explained by the passion construct. Moreover, an appropriate measurement model should account for the possibility that some individuals endorse only some of the facets, while others endorse all of them, and thus, we are also looking for measurement models that are able to reveal intra-individual co-endorsement patterns, which correlation- or regression-based models do not necessarily reveal (see Moeller et al., 2018a; Moeller et al., 2018b).

**Figure 3:**
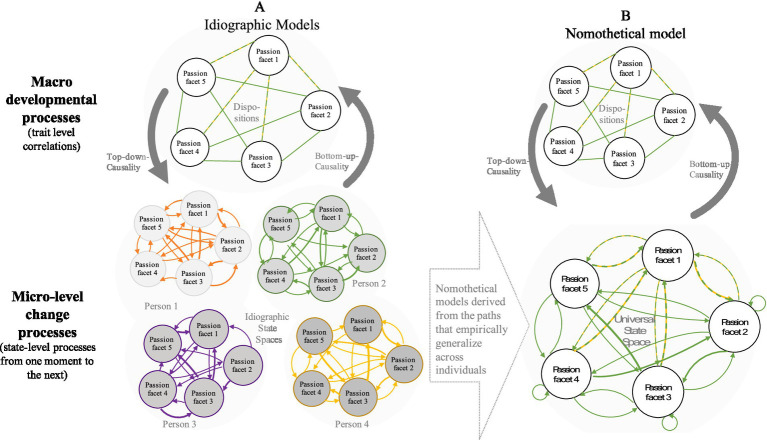
Multilevel integrations of intra-individual state and inter-individual trait relationships among passion facets in **(A)** a nomothetic approach and **(B)** idiographic approaches (model adapted for passion from Moeller et al., 2022).

In the research on other multi-facetted constructs, partial correlation network models have been proposed to measure multi-facetted constructs, including the psychological symptoms of psychological disorders (e.g., Fried et al., 2017; Pe et al., 2015), the interplay of personality facets (e.g., Costantini et al., 2015) or the relations among granular emotions (e.g., Lange et al., 2020). I propose that the psychometric network model discussed by Lange et al. (2020) and other network approaches meet the above-mentioned requirements for a measurement model representing crucial theoretical assumptions of the entrepreneurial passion construct. The psychometric measurement model assumes that the construct, passion, *is constituted* by the ensemble of its facets and by the (causal) relationships among these facets. It requires all relevant facets to be measured, requiring a comprehensive taxonomy of passion facets as its theoretical basis (which, e.g., Newman et al., 2019 have provided). The psychometric network model allows for the relationships among facets to vary within and between individuals (see the idiographic state networks in Figure 3). Psychometric network models further assume that the facets have causal influences on each other and that these interactions among facets are theoretically insightful and constitutive of the overall construct (entrepreneurial passion). This is a crucial feature that the psychometric network model offers beyond the commonly used structural equation models (formative or reflective measurement models).

Thus, a psychometric network model may be an appropriate representations of the assumed structure of entrepreneurial passion, because of the following reasons: (1) It allows to specify, measure, and disentangle the facets of entrepreneurial passion as distinct sub-constructs, (2) it allows for different relationships (correlations or regression coefficients) among different pairs of facets, (3) unlike the CFA and other formative measurement models, it does not represent the multi-facetted construct with one latent variable, thus does not insinuate that one entity or one mechanism has to *cause* the inter-correlations among the facets, and it thus allows for such “residual” correlations among facets that would otherwise would have led to a model rejection in CFA.

### Process models describing how situational experiences relate to stable traits

Specifying the facets of passion and representing them in psychometric network models can help us realize a third goal: Understanding how fluctuating states relate to rather stable motivational dispositions in people with an entrepreneurial passion. We already established above that many definitions and measures of passion include on one hand fluctuating facets known to vary from one moment to the next, such as flow, emotional states, or specific action plans (e.g., Forest et al., 2011; Vallerand et al., 2003; Moeller et al., 2017), and on the other hand facets considered as rather stable, such as inclinations, identification, long-term goals, or personality trait facets, including perseverance/grit (e.g., Jachimowicz et al., 2018a; Moeller et al., 2017; Tosun and Lajunen, 2009; Balon et al., 2013). But how do such fluctuating and rather stable facets play together? Do situational passionate experiences (e.g., rewarding experiences of success, competence, joy, flow) lead to the development of trait-like motivational inclination toward a given entrepreneurial activity (e.g., well-developed personal interest, long-term goals, dispositional task values or competence beliefs)? Or are people born with a disposition for a certain passion for a certain entrepreneurial activity, and this disposition just makes it more likely for them to experience motivational states such as flow and positive state emotions during such activities? Or is this passion just the label that we apply to name the overall interaction of a person with an activity in a given situation?

Several theories about related motivational constructs assume that motivational states may lead to the development of motivational traits, which might suggest that similar processes might apply to the development of entrepreneurial passion. For example, the four-phase model of interest development (e.g., Hidi and Renninger, 2006) assumes that repeated experiences of the fluctuating situational interest may lead to a well-developed personal interest, which is a rather trait-like disposition (e.g., Hidi and Renninger, 2006). Similarly, the dynamic expectancy-value model (Moeller et al., 2022; see also Eccles and Wigfield, 2020) assumes that rather stable competence beliefs and task value evaluations may develop out of repeated situational experiences. We do not know yet for sure if similar state-to-trait developmental processes contribute to the development of an entrepreneurial passion, because most studies on passion are rather cross-sectional than longitudinal, and few examine the presumably fluctuating and the presumably stable facets in joint studies.

However, the idea of development from states to the traits is supported by several theoretical reasons: first, passion is very similar to well-developed personal interest, even causing some researchers to call passion “the end of the interest continuum” (Gagné, 2007, p. 99). Due to large overlaps of interest with passion, we might adapt the four-phase development of interest from situational to well-developed interest to describe the development from passionate state experiences to a manifest and rather stable entrepreneurial passion.

Further reason supporting such assumed state-to-trait development and state–trait interactions can be found in system theories of personality (Mischel and Shoda, 1998) and the person-situation debates in personality research (e.g., Funder, 2006; Kenrick and Funder, 1988; Roberts and Caspi, 2001). These theories describe dynamic state–trait relations, including the possibilities that traits change through repeated situational experiences (Newcomb et al., 1967; Kenrick and Funder, 1988) and that the behavior and experience in a given situation is determined by both stable and malleable aspects (Fleeson, 2004; Funder, 2006; Mischel and Shoda, 1998; Kenrick and Funder, 1988). Network-like systems of interacting personality facets were described as early as 1995 (Mischel and Shoda, 1998) and have seen a recent boom in numerous articles on the network relationships among personality facets (e.g., Costantini et al., 2015, 2019; Fonseca-Pedrero et al., 2018).

A link between these theories of personality development and an entrepreneurial passion can be drawn due to the trait-like passion components that have been described in several models and studies on both entrepreneurial passion and passion for other activities (e.g., Balon et al., 2013; Cardon et al., 2009; Duckworth et al., 2007; Moeller, 2014; Moeller et al., 2017; Obschonka et al., 2019; Tosun and Lajunen, 2009). If we assume that a part of passion is a rather stable inclination, meaning a trait-like tendency to act in a certain way, then we may can draw parallels from the personality development research about the possible development of such traits, and their relationships to specific affective and motivational states. Some of the insights from the person-situation debate that may help moving the research on entrepreneurial passion forward are: (1) stable and malleable facets interact in specific situations, (2) these interactions are determined by characteristics of the situation and context, malleable and stable characteristics of the person, and specific interaction mechanisms (e.g., Mischel and Shoda, 1998; Funder, 2006), (3) dynamic systems models help understanding the interplay of affective and cognitive state and trait components (e.g., Mischel and Shoda, 1998), (4) within-person variability must be studied to understand the interplay of fluctuating and stable determinants of behavior (e.g., Fleeson, 2004), and (5) individuals vary in their intra-individual structure and processes among such personality facets (e.g., Mischel and Shoda, 1998).

**Figure 4:**
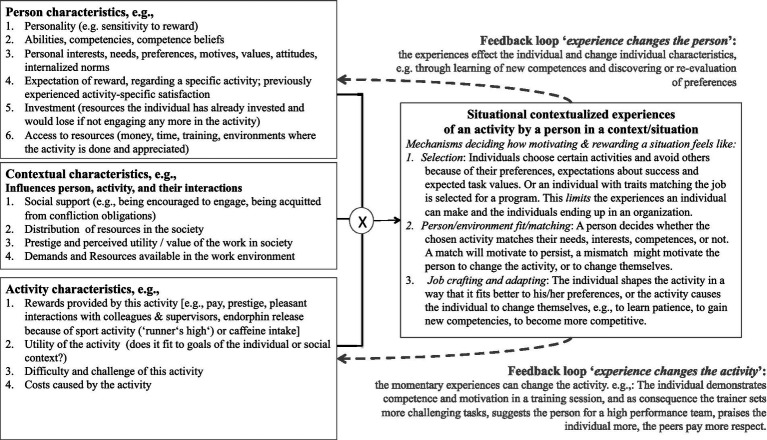
Pathways of possible interactions between person-specific and activity-specific determinants of passion (adapted from Moeller, 2014; see also an adapted version for entrepreneurial passion in Newman et al., 2019).

Figure 3 proposes a multilevel model integrating these insights from the person-situation personality debate with the facet and network approaches for research on entrepreneurial passion. The specific contents of possible interactions among specific state and trait components are depicted in Figure 4. The multilevel model of Figure 3 defines entrepreneurial passion as the ensemble of passion facets, whose inter-relations are depicted in network models. The lower networks in Figure 3 represent the intra-individual moment-to-moment dynamics among facets, based on variance between situations, within individuals. The upper networks represent the cross-sectional, presumably stable inter-individual correlations among traits, based on variance between individuals.

This multilevel model describes various processes of change: (1) The moment-to-moment predictions among passion facets on the state-level (lower networks), (2) the state-to-trait development (arrows from the state- to the trait-level; bottom-up causality) and (3) trait-to-state influences, (arrows from the trait to the state level; top-down causality).

Figure 3 comes in two panels: The idiographic version (model A) on the left allows for differences (heterogenity) between individuals in regard to the intra-individual structure and processes among passion facets. The latter is in line with the heterogeneity described in Mischel and Shoda (1998) and in the recent literature on idiographic networks and intra-individual analyses (e.g., Beck and Jackson, 2020). The nomothetical version (model B) on the right assumes that one intra-individual model appropriately describes the intra-individual processes and structure among passion facets in the sample. I will address the reasons for including such idiographic models more in detail below.

All of the assumed developmental processes depicted in Figure 3 (moment-to-moment, state-to-trait and trait-to-state) yet have to be tested for entrepreneurial passion. A useful method for such empirical tests would be the experience sampling method (ESM; e.g., Larson and Csikszentmihalyi, 2014). ESM is typically used to capture fluctuating experiences, such as state emotions, flow, or short-term goal setting (Ketonen et al., 2018). So far, ESM has been used rarely in studies on passion (e.g., passion in adolescents, Moeller et al., 2017), or studies on related constructs, such as work engagement and workaholism (Snir and Zohar, 2008). In order to combine the state assessments of ESM studies with the long-time perspective needed for studies of traits, future studies could combine intensive short-time period of data collection (e.g., 1 week of intensive experience sampling) with a longer time span capturing the possible emergence of henceforth rather stable motivational dispositions (e.g., with measurement bursts in the same individuals a few months or years apart, Moeller et al., 2017).

While Figure 3 aims to illustrate the multiple levels of analysis and the time lines of development to help integrating state and trait aspects in joint *research designs*, we need more specific hypotheses about the mechanism that we expect to link state-to trait aspects of passion. Such specific hypotheses are depicted in Figure 4, which builds upon the personality trias (situation, person, behavior/experience) described by Funder (2006) and the interactions between situational, personal, contextual, and interactional determinants described in the person-object theories of interest (e.g., Krapp, 1998; Moeller et al., 2020).

Figure 4 shows which situation characteristics and person characteristics are expected to predict the experience of passionate states in specific situations. An important feature of this model are the feedback loops, processes through which the situational experience of passion facets changes the person or the situation characteristics of future experiences. Through these iterative feedback processes, the situational experiences of passion-related facets in specific contexts and moments are outcomes and predictors, of person and situation characteristics.

Such feedback processes (e.g., personality traits influencing state experiences and state experiences influencing personality traits) are assumed by some authors of the person-situation debate in the personality literature (e.g., Mischel and Shoda, 1998; Kenrick and Funder, 1988) and they are in line with the idea that passion facets can influence each other mutually (see the lower networks in Figure 3 and the network model 2 in Figure 1). Feedback processes also help us solve the apparent contradiction that some constructs, such as flow or positive emotions, are alternatingly described as predictors and outcomes of passion in previous studies. By describing feedback processes, we gain an understanding of mechanisms allowing us to consider flow and positive emotions to be both predictors and outcomes of other passion facets. A further advantage of feedback processes is their ability to describe self-reinforcing processes (vicious circles or virtuous circles, depending on their desirability). Figure 4 shows feedback processes that seem plausible in the development of entrepreneurial passion, such as the feedback loop from the state experience to the person (person develops skills, or preferences, or expectations about own success and future reward) or the feedback loop from the state experience to the activity or context characteristics (e.g., increasing challenges, increasing social support by colleagues, supervisors or mentors, promotions leading to a new work environment or new work tasks, in- or decrease in rank and reputation).

One such self-reinforcing mechanism may the experience of reward or incentive salience, which by definition raises the likelihood of a re-engagement and a desire for more (e.g., Berridge, 2007). The experience of rewarding emotional and motivational states (positive state emotion, feeling of success, flow) may motivate the person to re-engage in similar activities in the future in the hope to experience similar reward again. This, in turn, may lead over time to gains in expertise and achievement, positive feedback from supervisors or colleagues for showing up, spending extra time, getting more done, radiating a positive attitude, etc. Such praise, in turn, is likely to be experienced as a rewarding state, which in turn may re-inforce the motivation to engage in similar activities in the future. Over time, we can imagine such a virtuous circe to culminate in an awareness of the individual that this activity is really “their thing,” entailing processes of increasing identification with and fondness of the activity that has provided so many rewarding experiences. The involvement of the reward may explain the overlap of (obsessive) passion with concepts like workaholism and obsession (Birkeland and Buch, 2015; Gorgievski-Duijvesteijn and Bakker, 2010).

### Dialectical models of ambivalent motivation: the need for intra-individual methods

As stated above, both positive, intrinsic, and negative, aversive feelings and experiences can accompany passion, sometimes even in the same individuals. Likewise, the assumed predictors of HP and OP, intrinsic and extrinsic motivators, can co-occur in the same individuals (Lepper et al., 2005). While it has been an important first step to integrate both aspects in one joint model (Vallerand et al., 2003), it appears that the dualistic interpretation of HP and OP as almost mutually exclusive experiences, or as distinct types or groups of individuals (e.g., Vallerand et al., 2003; Vallerand, 2012) does not do justice to the ambivalent nature of passion (Moeller et al., 2019) and of other forms of work motivation (e.g., exhaustive engagement, see Moeller et al., 2018b). I therefore propose a *dialectical understanding of passion as a form of ambivalent motivation* by including the following assumptions into definitions, measures, and study designs of entrepreneurial passion:

#### Statement 1

Entrepreneurial passion may be accompanied by positive, enjoyable and negative, aversive feelings and experiences. These feelings and experiences can co-occur in the same individuals, maybe even the same situations, which we shall call ambivalent motivation and interpret in reference to the research on mixed emotions (e.g., Larsen and McGraw, 2011; Hoemann et al., 2017) and other forms of ambivalent work motivation (e.g., exhaustive engagement, see, e.g., Moeller et al., 2018b). In other cases, a person’s passion can be characterized by predominantly positive experiences, which we shall continue to call an HP, or by predominantly negative feelings, which we shall call an OP, in line with the distinction in the dual model of passion (Vallerand et al., 2003).

#### Statement 2

We need intra-individual analytical methods to discover the intra-individual co-occurrence of positive and negative experiences and feelings in individuals with an entrepreneurial passion. Such methods can include analyses revealing intra-individual profiles (e.g., cluster analysis, latent profile analysis), methods revealing co-endorsements (e.g., co-endorsement network analysis, see Moeller et al., 2018a; Kulakow and Moeller, 2024; Jähne et al., 2024), or sometimes the simple visual inspection of scatter plots, with positive experiences on one axis and negative experiences on the other axis, to discover or rule out groups with ambivalent motivation (see, e.g., Moeller et al., 2015). It is important to keep in mind that correlation- or regression-based methods, both inter- and intra-individual ones, may obfuscate subgroups showing unexpected profiles (Anscombe, 1973; Matejka and Fitzmaurice, 2017; Moeller, 2021), which may lead to ambivalent motivation being overlooked unless scatter plots and the intra-individual profiles are inspected. Likewise, we should keep in mind that even the strongest correlations or regressions do not necessarily reveal or imply that two constructs are experienced together (because the co-variance may be driven by the individuals who did not endorse the relevant items), so that intra-individual co-endorsement analyses (e.g., Moeller et al., 2018a) are needed for conclusions about the co-occurrence of two psychological experiences in the same person, the same situation, or the same team or organization. For these purposes, it would seem a promising avenue for the hitherto mostly inter-individual passion research to embrace the intra-individual methods which are currently seeing a boom of innovation and debate in many other fields of social sciences, including many studies on motivation and emotions in achievement settings (e.g., Beck and Jackson, 2020; Brose et al., 2020; Völkle et al., 2014; Molenaar, 2004; Murayama et al., 2017; Reitzle and Dietrich, 2019).

#### Statement 3

We do not yet know much about the dynamics in the relationships among these positive and negative experiences over time. We need longitudinal studies to find answers to questions such as: can a person’s entrepreneurial passion develop from a harmonious one into a more obsessive passion if for example the circumstances and entrepreneurial activities change (e.g., think about a startup founder who loves her job but then faces extreme stressors and goal conflicts when a pandemic hits, or about an entrepreneur who is harmoniously passionate except for the time leading up to an important deadline, causing increased work time, stress, and conflicts with other goals in life, such as family-related goals or goals related to other entrepreneurial activities)? If a HP can turn into an OP, and maybe an OP into a HP, can we actively change a person’s passion (think about a manager or sports coach who sees the risk for exhaustion and wishes to help their employees/athletes get back from an OP into a HP)? On what time line do individuals who report ambivalent passion experience positive and negative feelings? Do those ambivalent experiences occur in the same situations, or sequentially, and what are the implications of one or the other? What are the predictors, correlates, and outcomes of such ambivalent passion? How sustainable is such ambivalent passion, for instance, is it a pathway from a HP turning into an OP or vice versa?

At this point, we can only speculate, because previous research has neither provided enough long-term longitudinal studies on the stability versus variability of passion over time, nor has previous research considered that individuals with a higher HP might become individuals with a higher OP and vice versa (leaving apart the findings that most individuals have a higher HP than OP and that the idea of classifying individuals into types with distinct HP-OP-profiles was merely a theoretical idea and not very well supported by the methods used and the findings obtained, see, e.g., Moeller et al., 2015). Future longitudinal studies could examine in what time frames long-term changes in passion might occur; whether, for instance, changes are likely to happen over weeks, months, or years, or hat specific stressors or contextual changes might trigger these shifts. Expanding on these questions would add richness to the temporal dimension of passion research.

#### Statement 4

In a next step, intervention studies could examine which facets can be influenced by managers, colleagues, or employees themselves, and which facets are less malleable. Without such knowledge, managers can only select the individuals to their organization, or select their behavior toward employees, based on the employees’ passion, but maybe managers may want to know how to optimize their employees’ passions, how to increase it or how to turn an OP into an HP, if possible. For such aspirations to be evidence-based, we need intervention studies. Distinguishing between the malleable and the less malleable facets may help us find out which facets should be targeted in such interventions.

### Integrating processes of striving and thriving

The here proposed conceptualization of entrepreneurial passion as facet-specific, possibly ambivalent, and consisting of processes running on different time lines, may help solving the puzzle of enthusiastic perseverance in effortful and partially aversive courses of action, which seems particularly relevant for the research on entrepreneurial passion, given that entrepreneurship typically involves risk, frustrations, setbacks and a need for perseverance in the face of obstacles.

Nearly every motivational theory can explain why people engage and persist in activities that are fun, perceived as valuable, interesting, in line with personal goals and identities. In other words, many motivational theories can explain motivation as long as the motivated choice is a rational and/ or a hedonistic choice. Such rational choice or hedonistic explanations would suggest that individuals stop feeling motivated and should give up on an activity or a work task as soon as it becomes too costly, too adverse, or too painful.

Other constructs describe why people persist through adverse, effortful, or painful periods. Those constructs mainly emphasize the ability to cope and regulate the negative emotional consequences of such adversity (e.g., self-regulation) or the power of principles, willpower, and sheer stubbornness, all of which can be considered ways of subduing one’s feelings and ways of putting rational considerations above the affective motivational drivers.

The interesting feature of passion, however, lies in its ambivalence. Instead of just enduring the adverse periods, we see individuals who despite of all the suffering claim passionately that they cannot imagine doing any other work than this, who describe an urging desire to go back and do the same entrepreneurial activity again, individuals who seem to *persist not despite but because of their feelings toward the activity*. This may be the unique niche, or research desideratum, that passion may fill like no other motivational construct so far.

At first, it appears paradoxically, how can someone report such an urging desire and positive evaluation of an activity which may be extremely costly, dangerous, and even painful, all the while reporting strong negative feelings about that activity? What exactly are their motivational drivers? The answer may lie in the concepts introduced above, particularly the dynamic interplay of different facets over various time spans. A passion for entrepreneurial activities is expected to be driven by positive affective motivational forces, but also expected to persist in the absence of such positive drivers. These may be two different motivational mechanisms, emotional thriving and effortful striving, that may come together or follow each other subsequently. To understand their interplay over time, I propose a distinction between the motivational forces that energize approach motivation and enthusiasm (positive emotions, flow, and the aspects of activities that are experienced as being fun and autotelic), on one hand, and the motivational forces that allow individuals to endure in the absence of autotelic experiences (grit, long-term goals, self-regulation, self-control skills, and the mixture of unfazed indifference, stubbornness, and determination that Finns call sisu; e.g., Lahti, 2013), on the other hand. The former be called thriving and the latter striving.

While each of these both sides can exist without the other, we can assume that a motivation is stronger and more resilient if they co-occur in the same individual. A person with a lot of willpower and grit may persist in an adverse activity (e.g., an entrepreneur developing a company after repeatedly being denied funding and acknowledgement, or an entrepreneur seeking new collaborators and participating in entrepreneurial contests after losing opportunities due to prior industrial espionage). Such a determined individual may burn out after a while, or may reconsider after a while if this costly activity was the most rational or sustainable choice after all, or if the persistence in the face of obstacles resembles an escalated commitment that should be ended. On the other hand, a person who is mostly motivated by strong positive feelings, frequent flow experiences, and autotelic, fun, gameful entrepreneurial experiences may want to quit once a dry spell or an extended phase of difficulties comes up. The sweet spot of motivational resilience, however, may lie in an individual’s ability to alternate between one and the other and to recalibrate the motivational strategy to changing environmental circumstances.

The fun parts may contribute the motivational energy, drive, approach motivation, and the ability to imagine better times even when the going gets tough. On the other hand, the long-term goals, grit/perseverance trait, and determination may carry across the dry spells, the times when an activity is difficult, frustrating, stressful, painful, or when nothing seems to be working and when none of the basic needs are met (e.g., when one feels incompetent, lonely, or ostracized, and constraint in one’s autonomy).

The assumed interplay of processes of striving and thriving is depicted in Figure 2 below, and Table 3 suggests which passion facet is hypothesized to cause engagement and persistence during which phase of this model. The important features of this model are (1) the distinction between passion facets that drive striving (e.g., grit, particularly the grit perseverance facet, identification, long-term goals) and passion facets that drive the emotional thriving (e.g., flow, enjoyment), (2) the assumed compensation mechanisms, with striving (persistence) compensating for a temporary lack of thriving (fun), with recovery compensating for the loss of resources through both the striving and thriving processes, and with thriving compensating for the lack of fun during striving phases, (3) the assumption that aspects of thriving and striving may co-occur and that this co-occurrence may be the reason for the sometimes observed ambivalence and mixed emotions, and (4) the addition of recovery processes, which may account for much of the sustainability in passionate entrepreneurial work (to paraphrase Arnold Bakker, who tweeted: “Being too engaged is asking for trouble. Engagement is a peak, and peaks need lows. For example, by psychologically detaching from work in the evening”; Bakker, 2018; see also the quote by Byron, 1833, at the beginning of this article).

**Table 3:** Which passion facets are expected to contribute to which strive and thrive phases.

	Paddling	Good weather surf	Stormy weather surf	Recovery
*Striving facets*	X		X	
Grit, conscientiousness	X		X	
identification	X		X	
Long-term goals	X		X	X
Action plans	X	X	X	X
Obsessive experiences	X		X	
Negative emotion (e.g., stress, frustration)	X		X	
*Thriving facets*		X	X	X
Desire/ approach motivation	X	X	X	
Harmonious experiences		X	X	X
Liking		X		X
Positive emotions (e.g., joy)		X	X	X
Recovery				X
Resources building				X
Goal re-evaluation				X
Flow		X	X	
Interest		X	X	

It should be noted that the distinction between thriving and striving resembles somewhat the distinction between pull and push motives described in regard to the Sport Commitment Model by Scanlan et al. (2009, 2010) and adopts the distinction between interest-driven factors and persistence-emphasizing factors in Duckworth et al. (2007) grit model. Thus, the strive and thrive model is proposed not in the hope to replace other models, but in the hope of helping to reconcile them, on the basis of the above-mentioned innovations (facet-specificity, network structure, feedback loops, state–trait distinction, and ambivalent motivation).

I hypothesize that striving and thriving are relatively independent processes that may co-occur or alternate in a passionate entrepreneur. Borrowing from the demands-resources model of work engagement and burnout (Bakker and Demerouti, 2007; Bakker et al., 2023; see also Wang et al., 2023 for links between entrepreneurial passion and the demands-resources model), I hypothesize that thriving may happen when an entrepreneur has enough resources (such as competences, funding, supportive, capable, and uplifting business partners or employees) to keep up with ongoing challenges. Striving, on the other hand, may occur when demands outnumber the resources and coping skills of the entrepreneur for an extended time, when setbacks happen that cannot be compensated, or when workaholism or other reasons lead to the depletion of recovery, leisure time, social support systems, and personal coping mechanisms. The demands-resources literature suggests that both the extend of demands and resources, and the balance between both factors, are deciding factors influencing strain (striving) and flourishing (thriving; e.g., Bakker and Demerouti, 2018). These hypotheses need to be tested for the Strive (S) and Thrive (T) Process Model of Entrepreneurial Passion. The fact that the demands-resources model was found useful to explain work engagement and work burnout (Bakker and Demerouti, 2007, 2018; Bakker et al., 2023), and the fact that both of these constructs have been linked to entrepreneurial passion (e.g., Wang et al., 2023), suggest that some of the insights from this literature may be transferable to the research on entrepreneurial passion.

As the empirical research on the demands-resources model has shown, too much strain can lead to burnout and depression in the long run. Thus, without the recovery, and without the positive experiences provided by the factors causing thriving, excessive demands, excessive perseverance and excessive motivated behavior come with risks (e.g., De Mol et al., 2018). On the other hand, if entrepreneurs rely exclusively on intrinsic motivation and positive emotions, they may miss the opportunity to train and brace themselves for eventual hardships and setbacks, and may be unprepared when these hit. I hypothesize that self-regulation skills, including goal adjustment, emotion regulation, and the anticipation of setbacks and required behavioral, cognitive and emotional responses, will be a core competence enabling entrepreneurs to experience thriving in the face of challenges and to down-regulate causes and outcomes of the striving processes. That is why the Strive (S) and Thrive (T) Process Model of Entrepreneurial Passion includes recovery, which is a crucial condition of successful self-regulation (e.g., Beckmann and Kellmann, 2004). Recovery is the hypothesized replenisher of personal resources, which in turn is expected to help the entrepreneur regain balance, regulate emotions and motivation, and recalibrate their problem-solving strategies.

### Cumulating insights by joining forces in multi-lab collaborations

Collaboration and interactive, cumulative discussion among passion researchers is much needed to develop a consensus about definitions and measurements of entrepreneurial passion. To achieve an integrative cumulation of insights across labs and across disciplines, the research it would be helpful to swap the currently prevailing practice of models, measures and studies being developed in silos, for collaborations across research teams to facilitating debate and integration. Table 4 gives an overview of open science practices that can be used to facilitate such collaboration among entrepreneurial passion researchers, along with available open science resources to build upon.

**Table 4:** Open science practices proposed to advance the research on entrepreneurial passion.

Open science practices	Specific examples and resources to build upon
1. Joint concerted efforts to launch debates among passion researchers about facets, definitions, and measures of entrepreneurial passion	a. Article collections or books in which different authors reply to each other’s points; with replies and counter-replies (for examples, see, e.g., debates about the generalizability crisis in Yarkoni (2022) or the debate about the role of dopamine in personality in Depue and Collins (1999)); b. Controversial multi-perspective expert panel discussions in conferences with subsequent transcription and publication of the debate among the various experts representing different standpoints (for examples, see the debate about the question whether we can “create gifted people” in Ericsson et al., 1993 or the positivism dispute in the German Sociology; Adorno et al., 1976) c. Reviews comparing multiple facets and features of different passion models (e.g., Iyortsuun et al., 2019; Lee and Herrmann, 2021; Moeller, 2014; Murnieks et al., 2020; Newman et al., 2019; Riar et al., 2023; Schwarte et al., 2023; Thongmanivong, 2020) d. Theoretical articles integrating the different facets of passion models in joint frameworks (e.g., Moeller, 2014)
2. Collections and overviews of passion measures and information about their psychometric properties on one joint open repository	a. Open item repositories, such as the international personality item pool (IPIP; e.g., Goldberg et al., 2006) or the experience sampling method (ESM) Item repository (Kirtley et al., 2018) b. Studies comparing different measures in the same sample, c. Meta-analyses comparing multiple measures (e.g., Zhao and Liu, 2023)
3. Multi-lab data collections	ManyLab study (Moshontz et al., 2018), the ManyBabies project, ManyBabies study (e.g., ManyBabies Consortium, 2020), ManyMoments study
4. Making existing data open for secondary data analysis	Open data repositories, such as the Open Science Framework (OSF; see, e.g., Tackett et al., 2019) or the Interuniversity Consortium for Political and Social Research (ICPSR) data repository (e.g., Lee and Jeng, 2019).

The goals of such collaborations can range from working toward a consensus on definitions and measures, to multi-lab data collections or joint secondary analyses of existing data, following examples of, for instance, the ManyLab project (e.g., Moshontz et al., 2018), the ManyBabies project (e.g., ManyBabies Consortium, 2020), or the ManyPrimates project (Altschul et al., 2019). Multi-lab collaborations could compare the incremental validity of various measures in large-scale surveys to figure out which model or which facet provided which insight, and which facet or measure was the best predictor for which outcome. Furthermore, collaborative studies could test the replicability of research findings across samples, or their generalizability across contexts (e.g., across entrepreneurial organizations, different entrepreneurial activities, phases of the entrepreneurial context, entrepreneurial domains, and other context and work attributes). These would be important steps toward the cumulating integration of various definitions, measures, and insights from various data sources in joint frameworks.

As specific measures facilitating cumulative research processes, I propose: (1) joint concerted efforts to launch debates among passion researchers about facets, definitions, and measures of entrepreneurial passion, (2) openly accessible item collections and overviews of their psychometric properties of various passion measures on one joint open repository, (3) multi-lab data collections, and (4) making existing data open for secondary data analysis.

*The first suggestion, joint concerted efforts to launch debates among passion researchers about facets, definitions, and measures of entrepreneurial passion*, could for instance be facilitated through conference panel or roundtable discussions, webinars, retreats, or hackathons, adopting the communication toolbox that used in the open science movement and entrepreneurship research (e.g., Chartier et al., 2018; Cristia et al., 2020; Irani, 2015). The technology and communication infrastructure is ready to be used (e.g., Crüwell et al., 2019). Joint debates of entrepreneurial passion researchers could be accompanied, or preceded, by (online) surveys asking these researchers, for instance, about the passion facets they would in- or exclude from the definition of entrepreneurial passion, and about their reasons for these suggestions. The goal of such a survey and subsequent in-person debate could be the development of a taxonomy of passion facets as a joint reference facilitating the later debate about boundaries and similarities among passion models and related constructs in the future. There are several examples in the literature of highly insightful write-ups of such past debates about controversial research topics, such as the publication summarizing the positivism dispute in the German Sociology in the 1970s (Adorno et al., 1976), or the written-up documentation of the panel discussion “can we create gifted people” from the 1993 CIBA foundation symposium (Ericsson et al., 1993). These previous examples suggest that an actual debate with the forth- and back exchange of arguments and suggestions can yield much insight that the currently prevailing publishing of separately working researchers hardly reaches.

Since entrepreneurship research is an interdisciplinary field, it seems appropriate to invite not only psychologists, but researchers and practitioners with interdisciplinary backgrounds to debates about facets, definitions and measures of passion. A dialogue with fields such as organizational behavior and leadership studies promises to enrich the research and our understanding of entrepreneurial motivation. Such cross-disciplinary collaboration could introduce new insights and methodologies that may not emerge from within the field of psychology alone.

The second suggestion, *joint collections and overviews of passion measures and information about their psychometric properties on one joint open repository*, can be reached by collaborating across research teams when sharing passion scales, other passion measures, and information about their psychometric properties. Currently, researchers who make their passion measures freely available do that for their own measures, often on their own websites or in appendices of more or less openly accessible publications. Easier access and better overview of existing passion measures could be provided by using the open repositories that the open science movement has established, such as an etherpad linked to a project page on the Open Science Framework, similarly to open item repositories such as the International Personality Item Pool (Goldberg et al., 2006) or the repository for Experience Sampling Method items (Kirtley et al., 2018). A repository could also link each measure to information about available translations by other than the original authors, and inform about current work on translations by any team.

These various measures should be posted on the repository accompanied by detailed information about their psychometric properties. In a next step, the various passion measures should be combined systematically in joint empirical studies, to compare findings (e.g., incremental validity) across measures and facets, to determine to what degrees and in which facets different measures overlap with of differ from each other. This would be a change from the current practice, in which most researchers use mostly their own favorite measure.

*The third suggestion, joining forces in multi-lab collaborations* could be implemented by researchers from various labs and locations gathering data with similar methods in parallel, enabling them to study the replicability across labs and samples, and the generalizability across contexts (e.g., organizations, work cultures, etc.). This may also make large-scale data collections possible at scale a scale that no individual team could have achieved, which could be used as a means to compare many different passion measures, and/or passion facets, in regard to their incremental validity in predicting relevant outcomes. In another approach, existing datasets including similar constructs could be pooled across labs for secondary data analyses. Such secondary data analyses could for instance re-analyze data with novel (e.g., intra-individual) methods or examine whether findings remain invariant across different entrepreneurial work contexts and samples or across different teams of analysts (see, e.g., Bastiaansen et al., 2019).

*The fourth suggestion, open data,* would facilitate the re-use and therefore the transparent and efficient use of research data, and would serve the merging of existing data across labs for joint secondary data analysis. Re-analyses of transparently and openly shared data would serve to check whether previously reported findings and conclusions remain robust robustness when different methods, such as the above-mentioned intra-individual analyses are used, or if such novel methods add novel insights. Pooling existing datasets across samples and labs also provides opportunities to examine whether findings remain invariant across contexts and cultures, or if systematic differences between these contexts reveal the existence of contextual boundary conditions. For instance, we might want to test the hypotheses that some entrepreneurial activities, roles, domains, jobs, or some periods in history, provide more reasons to feel obsessed and aversive about work than others, having in mind how healthcare workers feel during a pandemic, researchers before a deadline, athletes before important competitions, or entrepreneurs before important funding, rating, or acquisition or merging decisions. Following this example, we might have hypotheses about domain- and time-specific boundary conditions concerning the relations of obsessive passion and certain negative emotions with other passion facets and passion outcomes. A way to test this hypothesis would be to examine these relationships in large datasets including data from many different domains, or data from before and during the recent COVID-19 pandemic. While this might be difficult for a single lab, it might be fast and easy for a collaboration of many labs. Open data sharing can be done retrospectively (by making existing data available that publications were based upon via repositories such as the Open Science Framework; e.g., Tackett et al., 2019, or the ICPSR data repository; e.g., Lee and Jeng, 2019) and prospectively (by committing to making upcoming data available in the future, as soon as publications are accepted).

Research on entrepreneurial passion is research on innovation. It crucially needs to innovate itself by keeping up with new developments in research on motivation and other areas pf psychology relevant to passion, including personality psychology and current methodological innovations. In sum, I propose that this process of innovating research on entrepreneurial passion would benefit greatly from distinguishing systematically between passion facets, employing concepts and measures specifying the role of states and traits in entrepreneurial passion, exploring the ambivalent nature of passion, debating suitable and less suitable measurement models, using within-person methods, and cumulating research efforts in open science practices.

#### Practical implications for entrepreneurs

From the solutions proposed in this article, practitioners such as entrepreneurs or managers might gain practical benefits in the future. The fact that person-oriented methods increasingly find that it is rather common for individuals to experience both obsessive and harmonious passion together changes the take-home-message that any counselor or incubator staff might give to nascent entrepreneurs. Rather than telling entrepreneurs that they are either the obsessive type who has to worry, or the harmoniously passionate type who does not have to worry, the people in charge of providing motivational support, and the people making funding decisions, gain from the person-oriented research the insight that a highly passionate person is typically someone who is highly committed, highly enthusiastic, and yet at risk of being stressed, of overworking themselves, and in the worst case of burning out. Practical motivational support for passionate entrepreneurs should therefore include stress reduction, emotion regulation and burnout prevention services, as well as psychological education on these risks and how to spot and ameliorate them in oneself during the process of funding companies. The Strive (S) and Thrive (T) Process Model of Passion was developed to incorporate the insights about strain and necessary recovery that is part of passionate working processes. In contrast to previous passion assessments, which focused on stable, trait-like aspects of passion, this model reminds researchers and practitioners that strong motivation is a peak and that every peak needs its valley (i.e., recovery), an insight we share with research experts from the fields of work engagement and work burnout (Bakker, 2018; see also the quote by Byron, 1833, at the beginning of this article). Rather than telling entrepreneurs that they are the right (harmonious) or wrong (obsessive) type of passion, the Strive (S) and Thrive (T) Process Model of Passion reminds practitioners that aspects of their passion can change, and that sustaining a high motivation in a demanding field that requires you to keep on your tiptoes means to consciously assess the current resources and current strains and to actively balance them out.

## Discussion

This review article pointed out six challenges in the current research on passion for entrepreneurial and other activities, along with proposals for corresponding solutions (for an overview, see Table 1). The main goal of this review was to link research on entrepreneurial passion to current innovations and debates from other fields of psychology and related disciplines, including measurement and method development, emotion and motivation research, as well as personality and developmental psychology. Thus, the main goal of this review was to facilitate a cumulative learning process across psychological sub-disciplines. A crucial instrument toward a cumulative research process are open science practices, which promise to help us build bridges between research teams and sub-fields that without such collaboration risk to remain in isolated silos, unable to build on each other’s work. Improving the measurement of entrepreneurial passion and specifying the exact contribution of each facet to antecedents and outcomes will be crucial instruments for managers, founders, or employees who wish to effectively and reliably assess and foster passion in their organizations and work life. Elaborating on process models of passion and specifying the processes during which entrepreneurial passion contributes to thriving (effortless motivation) versus striving (effortful perseverance) will be crucial for practitioners who wish to use their own or their employees’ passion deliberately to increase motivated behavior in all phases of the sometimes enjoyable, sometimes challenging entrepreneurial process. Taken together, the six innovations proposed in this review promise to help researcher build bridges to each other’s work within and across sub-disciplines and to practitioners.

## Footnotes

^1^Thus, the goal of this review is to connect research on entrepreneurial passion to innovations and debates in *other* psychological sub-disciplines. It is explicitly not the goal of this review to summarize or give an overview of the current state of research on entrepreneurial passion, since such overviews already exist (see, e.g., Iyortsuun et al., 2019; Lee and Herrmann, 2021; Moeller, 2014; Murnieks et al., 2020; Newman et al., 2019; Riar et al., 2023; Schwarte et al., 2023; Thongmanivong, 2020; Zhao and Liu, 2023). Connecting entrepreneurial passion research to these debates and innovations form other psychological sub-disciplines is important, because it promises to help improving how we measure and define entrepreneurial passion, how well we understand its development, and how well we understand its relations to antecedents and outcomes. For an overview of the expected relevance of the various interdisciplinary aspects suggested in this review (see Table 1).

^2^A psychometric network is a graph that visualizes partial correlations among a set of variables, with each bivariate distribution being controlled for the influence of all other variables in the network. In such a network, the lines (called edges) represent the effect sizes and sign (+/−) of these partial correlations and the circles (called nodes) being connected by these lines represent the variables being measured (e.g., facets of passion).

^3^Likewise, a zero-order correlation network represents the zero-order correlations among all variables, without any bivariate correlation being controlled for the influence of other variables (e.g., Jähne et al., 2024).

^4^A co-endorsement network consists of lines (i.e., edges) that visualize how often the two variables connected by that line have been endorsed (affirmed) together, which is not the same as the information revealed by correlations (see, e.g., Moeller, 2021; Moeller et al., 2018a).

^5^Reflective latent variable models include a latent variable that represents the sole cause of inter-correlations among its indicator items, which are typically considered exchangeable and must not show any residual correlations. The commonly used confirmatory factor analyses are reflective latent variable models. For an illustration, see Figure 1.
